# Clinical Gray Zones of Cardiac Troponin Interpretation in the Emergency Department: When Increased Concentrations Do Not Equal Acute Coronary Syndrome

**DOI:** 10.3390/jcm15124444

**Published:** 2026-06-09

**Authors:** Johannes Mair

**Affiliations:** Department of Internal Medicine III—Cardiology and Angiology, Medical University of Innsbruck, Anichstrasse 35, 6020 Innsbruck, Austria; johannes.mair@i-med.ac.at; Tel.: +43-512-504-24118; Fax: +43-512-504-22767

**Keywords:** cardiac troponin, high sensitivity, emergency department, algorithm, acute coronary syndrome, acute myocardial infarction, confounder, myocardial injury

## Abstract

The introduction of rapid, high-sensitivity cardiac troponin (hs-cTn)-based algorithms has markedly changed the work-up of patients admitted to the emergency department (ED) with suspected acute coronary syndrome (ACS). However, when applied to real-world ED populations, these algorithms perform worse than in clinical studies of derivation and validation. The main reasons for this discrepancy are that patients tested for hs-cTn in real-world settings tend to be older and less clinically preselected. Nevertheless, ACS must often be ruled out in patients with atypical presentations. Routine patients also more frequently have impaired renal function and pre-existing cardiac diseases, such as atrial fibrillation, heart failure, or coronary artery disease. These conditions do not necessarily cause the actual acute ED presentation. Using the standard decision limits of the 0 h, 0/1 h, or 0/2 h algorithms does not hinder the exclusion of ACS in the ED. However, using them in real-world conditions substantially decreases the positive predictive value for acute myocardial infarction (AMI) and classifies a higher percentage of patients into the “observe (gray) zone” than reported in clinical studies. Patients classified with a working diagnosis of “rule-in AMI” often require hospital admission for other reasons, though their discharge diagnosis may differ from AMI. A major challenge in real-world EDs is the high proportion of gray zone hs-cTn concentrations in approximately 50% of tested patients. Therefore, additional hs-cTn sampling at 3 h after admission is often necessary to rule out acute myocardial injury. This review summarizes and critically discusses the evidence for adjusting hs-cTn ED algorithm decision limits according to age, sex, and renal function. It also discusses the critical differential diagnosis of acute and chronic myocardial injury in the ED.

## 1. Background

**Figure 1:**
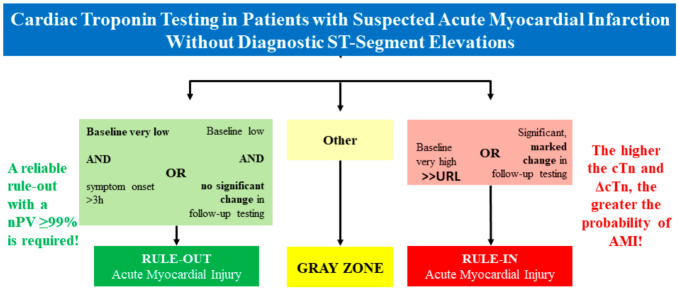
Rapid troponin-based algorithms for evaluating emergency department patients with suspected acute coronary syndrome. Abbreviations: negative predictive value (nPV), acute myocardial infarction (AMI), upper reference limit (URL), cardiac troponin (cTn), troponin concentration change in serial testing (ΔcTn).

Patients presenting to the emergency department (ED) with symptoms consistent with a diagnosis of acute coronary syndrome (ACS) are very common. Additionally, ACS must often be ruled-out in patients with less typical symptoms, such as shortness of breath, fatigue, nausea, dizziness, or fainting, particularly in women or elderly patients. However, only a minority of patients initially clinically suspected to have ACS will have a diagnosis of acute myocardial infarction (AMI), which complicates the rapid evaluation of these patients. Testing for cardiac troponin (cTn) is a cornerstone of the diagnostic approach. An international consensus published in 2000 [1,2] defined cTn testing as the new criterion standard of laboratory testing for myocardial injury. Former professional guidelines [3,4,5] have recommended cTn testing at presentation and again 3–6 h later, with additional testing after 6 h for patients with ECG changes or other clinical risk factors indicating intermediate or high risk. Over the past decades, the analytical sensitivity and precision of cTn assays at their low measuring range has steadily improved. This has resulted in the introduction of so-called “high-sensitivity” cTn (hs-cTn) assays into routine use [6,7]. The widespread adoption of these assays has enabled the introduction of rapid hs-cTn testing based algorithms for evaluating ACS in structured ED decision pathways (see Figure 1) after their derivation and validation in large clinical studies of patients with clinically suspected ACS [7,8,9]. This has markedly changed the routine work-up of ED patients with clinically suspected ACS. However, when applied to real-world ED populations, the performance of these algorithms is worse than in clinical studies, highlighting their limitations. This review summarizes and critically discusses the evidence for adjusting hs-cTn decision limits in specific clinical settings, as well as challenging clinical differential diagnoses of acute and chronic myocardial injury in ED patients.

According to the current consensus, the negative predictive value of rule-out algorithms should be ≥99%. It must be emphasized that these algorithms only rule out acute myocardial injury, not significant coronary artery disease. Depending on the pretest probability, subsequent imaging is mandatory. Regarding rule-in patients, the positive predictive value for AMI should be ≥70%. However, it must be emphasized that primarily acute myocardial injury is diagnosed, and AMI can only be diagnosed in the clinical context of myocardial ischemia as the cause of myocardial injury. There are numerous causes of myocardial injury, and most of these patients need hospital admission. When applied to a real-world ED population, gray zone patients who require additional testing usually predominate.

## 2. Limitations of Guideline-Recommended, Troponin-Based Rapid Algorithms for Evaluating Patients with Suspected Acute Coronary Syndrome in the Routine Emergency Department

There are many reasons for the discrepancy in the diagnostic performance of guideline-recommended, hs-cTn-based rapid algorithms (see Figure 1) for evaluating patients with suspected ACS in studies and in real-world use. Most importantly, real-world ED patients are, on average, markedly older (the average age of study populations is 60–65 years), and less clinically preselected (the average prevalence of AMI is 5–10%, versus 10–15% in clinical studies) [10,11,12,13]. Real-world ED patients more frequently have chronic renal failure (average creatinine concentrations within the normal range in clinical studies) and pre-existing chronic cardiac diseases [10,11,12,13]. In daily practice, ACS must also frequently be ruled out in patients with atypical symptoms, such as shortness of breath, fatigue, or nausea, especially when combined with upper abdominal symptoms, falls, or syncope, and particularly in women or elderly patients, before discharge from the ED is considered. Overall, this leads to a substantial increase of gray zone hs-cTn concentrations (approximately 50%) and a markedly reduced positive predictive value for acute myocardial infarction (AMI) in patients classified as “ruled-in AMI” in real-world applications [14,15,16,17,18,19,20,21,22]. Only a minority of patients tested for hs-cTn will have a non-ST-segment elevation AMI, which makes the rapid evaluation of these patients challenging. Therefore, a more appropriate term for patients who are ruled in by these algorithms is “ruled-in of acute myocardial injury” (see Figure 1), as AMI can only be diagnosed in the clinical context of myocardial ischemia, as evidenced by ECG findings or imaging [6,7,8,9]. There are numerous other causes of acute myocardial injury (see Table 1).

**Figure 2:**
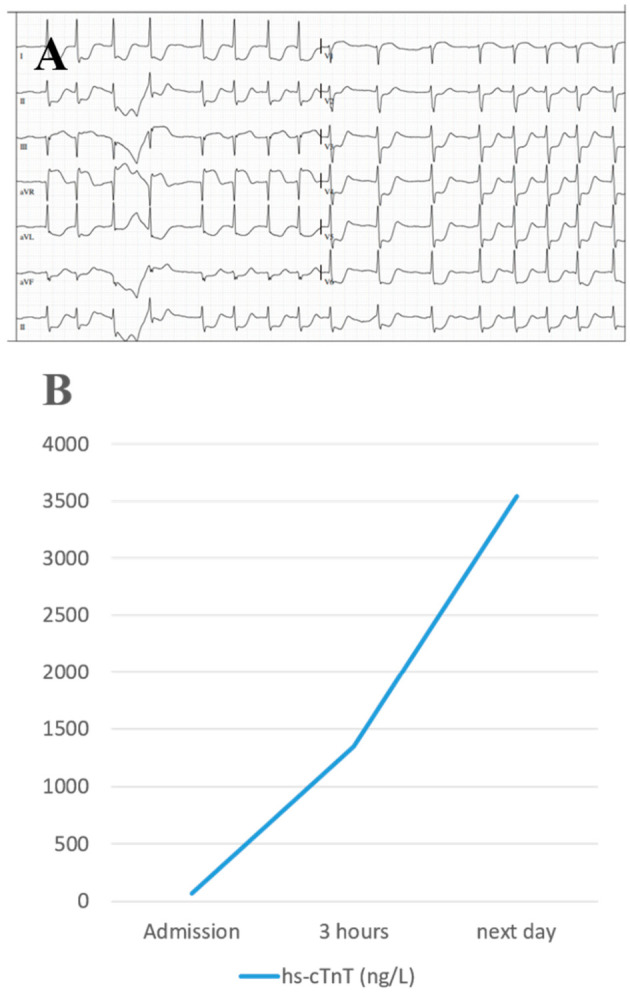
A clinical case demonstrating the challenges of interpreting troponin concentrations in patients with severe brain injury. (**A**) Admission ECG; (**B**) High-sensitivity cardiac troponin T (hs-cTnT) time course.

However, the majority of ED patients with acute myocardial injury need to be admitted to the hospital anyway, which is essential for rapid triage decisions. The most important causes of acute myocardial injury are AMI, other acute cardiac diseases (e.g., decompensated heart failure or symptomatic atrial fibrillation), acute pulmonary embolism, and acute brain injury (e.g., hemorrhage, ischemic stroke, traumatic brain injury, leading to acute myocardial injury). The latter conditions lead to acute myocardial injury via massive central nervous system activation of the sympathetic nervous system (see Figure 2) [23]. Additionally, despite enthusiastically rapidly ruling out ACS, other severe diseases presenting with chest discomfort that require further work-up and/or hospitalization may not be overlooked.

A 50-year-old man received bystander resuscitation following a cardiac arrest. On the emergency doctor’s arrival, ventricular fibrillation was detected and successfully defibrillated. Upon admission, the ECG revealed marked repolarization abnormalities in nearly all leads with ST-segment elevation in aVR (A). The admission hs-cTnT was moderately increased (71.4 ng/L, normal range < 14 ng/L) followed by a subsequent marked increase (B). An acute coronary angiography ruled out significant coronary artery disease. A subsequent cerebral computed tomography (CT) scan revealed a severe subarachnoid hemorrhage from a large aneurysm of the left posterior cerebral artery. The massive central nervous sympathetic system activation associated with the very severe subarachnoid hemorrhage led to secondary acute myocardial injury with marked repolarization abnormalities on the ECG and troponin increase. The patient died on the day after admission. In unclear cases of resuscitated patients, a cerebral CT scan is therefore strongly recommended prior to acute coronary angiography in order to rule out such “pitfalls”.

**Figure 3:**
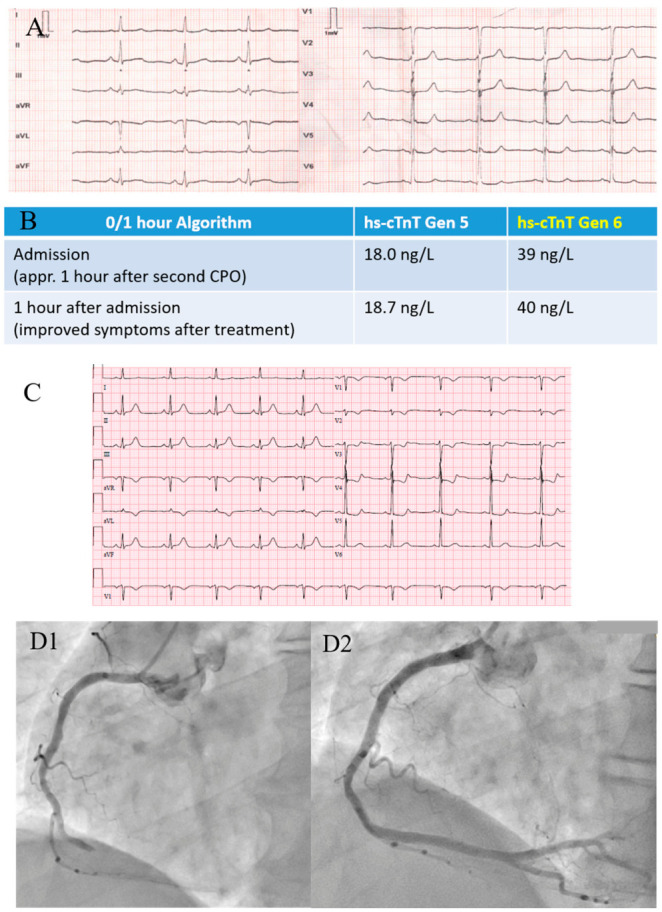
A clinical case illustrating the importance of considering all available clinical data when interpreting troponin test results—pitfall acute myocardial infarction with very early presentation. (**A**) ECG recorded by the emergency doctor; (**B**) hs-cTnT time course within 1 h from hospital admission; (**C**) ECG recorded 1 h after admission; (**D**) Right coronary artery before (**D1**) and after (**D2**) percutaneous coronary intervention. Abbreviations: approximately (appr.), chest pain onset (CPO), high-sensitivity cardiac troponin T (hs-cTnT), generation (Gen).

Using these rapid algorithms to rule out ACS also works well in a real-world ED [21], although it does not always achieve a negative predictive value of greater than 99% for predicting short-term major cardiac events [16,17,19]. The rule-out of AMI is often easier than the rule-in, provided that more than 3 h have elapsed since symptom onset without persistent symptoms and with a normal ECG (see Figure 3) [21].

This patient, who had a history of coronary artery disease (an anterior ST-segment elevation myocardial infarction in 2019 and an inferior non-ST-segment elevation myocardial infarction in 2022) experienced transient, brief, typical symptoms of angina at 1 p.m. while at work. He was symptom-free during his break, but experienced angina again at 5 p.m. with restarting work. The emergency doctor was alerted. The recorded ECG (A) showed only minor nonspecific ST-segment changes. Cardiac troponin T testing using the 0/1-h algorithm (B) did not indicate acute myocardial infarction, but it showed a mild increase in cardiac troponin T (including the latest assay generation for measurement) without a significant change within 1 h. Due to his high-risk clinical presentation, a transthoracic echocardiography was performed upon admission. This revealed a mildly reduced global left ventricular systolic function with anterior septal, inferior apical, and inferior lateral hypokinesia, as well as inferior septal akinesia. Given his history of previous myocardial infarctions, these findings were also not diagnostic. Due to improved, but still ongoing symptoms upon treatment, the ECG was repeated (C), demonstrating typical signs of acute myocardial ischemia (ST-segment elevation in the inferior leads and ST-segment depression in the anterior leads). An acute coronary angiography demonstrated thrombotic occlusion of the mid-right coronary artery as the infarct-related lesion (type C lesion with ruptured plaque, D1). This lesion was successfully reopened with primary percutaneous intervention (D2). This case demonstrates that, although all single diagnostic methods have limitations, a comprehensive clinical assessment can correctly diagnose an ongoing acute myocardial infarction with a very early presentation to the emergency department.

**Figure 4:**
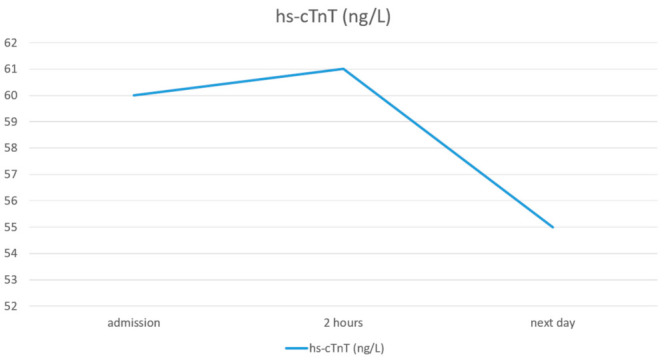
A clinical case demonstrating the importance of considering all available clinical data when interpreting troponin test results—pitfall acute myocardial infarction with late presentation. Abbreviations: high-sensitivity cardiac troponin T (hs-cTnT).

There are two different approaches: a single sample rule-out algorithm that uses substantially lower thresholds (usually values around the limit of detection of an hs-cTn assay) than the upper reference limit (URL), and a 0/1-h or 0/2-h approach based on values within the URL with a very low change in serial testing (see Figure 1) [21]. However, achieving a negative predictive value of at least 99% for AMI and a 30-day mortality for early discharge from the ED without further work-up in clinical routine also requires including all available clinical data for this decision (e.g., using the HEART score) [16,17,19]. When using an algorithm that incorporates a single hs-cTn value, the cTn test result must be obtained at least 3 h after the onset of symptoms without ongoing or recurrent symptoms [2,3,4]. It should be stressed that only acute myocardial injury can be ruled out, not significant coronary artery disease (CAD). Depending on the pre-test CAD probability, subsequent imaging is mandatory to rule out hemodynamically significant stenoses (see Figure 4) in order to achieve the published low event rate during short-term follow-up of clinical studies. However, it may be difficult or impossible to organize and ensure the necessary additional work-up outside regular working hours.

A 60-year-old man who had experienced angina pectoris the previous day presented to the emergency department with exertional dyspnea. On admission, his blood pressure was markedly elevated (180/95 mmHg). He had a history of arterial hypertension and diabetes mellitus type II (managed with oral medication). His standard admission 12-lead ECG was unremarkable, showing no evidence of acute myocardial ischemia. The time course of high-sensitivity cardiac troponin T concentrations is shown in Figure 4. The admission value of 60 ng/L was moderately elevated (upper reference limit 14 ng/L), the 2-h value of 61 ng/L showed no significant change. Despite the lack of a kinetic profile, the overall assessment suggested an acute coronary syndrome the previous day, with a plateau phase of troponin release. Chronic myocardial damage was deemed unlikely. Bedside echocardiography revealed normal left ventricular systolic function, with hypokinesis of the apical anterior septal and inferior septal segments. Acute coronary angiography performed on the day of admission confirmed acute coronary syndrome. Ruptured plaque leading to high-grade stenosis was found in the proximal anterior descending coronary artery with intracoronary thrombus formation. This type C lesion was successfully treated with percutaneous coronary intervention.

A major challenge in the real-world ED is that approximately 50% of patients have gray zone hs-cTn concentrations [14]. Therefore, additional hs-cTn sampling at 3 h after admission is often necessary to rule out acute myocardial injury [2,3,4,24], given the many causes of chronic myocardial injury (see Table 2). These causes may not be related to the patient’s acute ED presentation (e.g., in a patient with severe chronic heart failure and pneumonia).

In summary, clinical decision pathways that use serial testing of hs-cTn at presentation (0 h) and 1 h to 2 h later provide accelerated recognition of acute myocardial injury and early risk stratification, though they have limitations when using uniform decision limits. Table 2 summarizes the clinical rationale and evidence for adjusting these limits in specific clinical scenarios.

## 3. Current Evidence for Adjusting Cardiac Troponin Decision Limits in Specific Clinical Settings

### 3.1. Sex-Adjusted Decision Limits in Rapid hs-cTn-Based Algorithms

Cardiac troponin could not usually be detected in apparently healthy individuals with the first commercially available cTn assays [2]. When cTn was detected in these individuals occasionally, it was unclear whether the test result reflected true cTn increases or were instead analytical outliers or caused by analytical interferences [25,26]. The introduction of the first hs-cTn assays changed this, so that cTn was detectable in more than 50% of healthy individuals; using the latest generation of hs-cTn assays, cTn can be detected even in almost all healthy individuals [6,7,8]. The reasons for the presence of detectable cTn in healthy individuals are still not fully understood, given the limited turnover of adult cardiomyocytes and the limited regenerative capacity of the human heart [27]. However, it appears that there is ongoing turnover of cTnI and cTnT with leakage from human cardiomyocytes because such a high rate of analytical false-positive results could be ruled out in these individuals [26,28]. The use of hs-cTn assays has also revealed that the URL differs markedly between men and women for most cTn assays [29], a finding that also holds true for cTnT when tested with the latest generation of the hs-cTnT assay [30]. One obvious explanation is the difference in heart mass between men and women, though sex hormones may be involved as well. Thus, for diagnosing chronic myocardial injury in stable outpatients, it seems reasonable to use sex-specific URLs instead of a uniform URL. This would decrease the proportion of false-negative results, particularly in young women, and reduce false-positive results in elderly men. However, there is a lack of published evidence showing the clinically relevant net impact of this approach over using a uniform URL.

This approach could also be useful for patients with suspected acute myocardial injury who present to the ED. With hs-cTn assays, changes in hs-cTn concentrations within the normal reference range but below the URL could signal cardiac ischemia and may warrant further evaluation with repeat testing with calculation of sex-specific change or “delta” hs-cTn assay-specific diagnostic thresholds. However, in several studies, a net clinical benefit over using uniform limits could not be demonstrated for either [31,32,33,34,35], probably because other confounders, such as age and renal function, are clinically more important. In those studies, the reclassification rate was low, and risk stratification could not be improved by using sex-specific thresholds for either [31,32,33,34,35]. One plausible explanation for this finding is that, on average, women suspected of AMI are 10 years older than men. The effects of age and renal dysfunction, among other comorbidities may neutralize the effect of sex on cTn (see below). Additionally, evaluating dynamic cTn changes in women with baseline concentrations above the single rule-out threshold may attenuate the effect of using a lower sex-specific URL for ruling in clinically suspected ACS.

Ethnicity has no significant effect on cTn URLs and the performance of the 0/1-h algorithm [22,36]. Although no consistent effects of Asian ethnicity on cTn URLs have yet been published [22,36,37], it is important to confirm the diagnostic performance of the cTn-based algorithms recommended in guidelines in populations that were not included in the multicenter trials used to derive them. This is because ED populations may also differ significantly depending on local healthcare systems. If adjustments are recommended based on local experience (e.g., increasing the baseline rule-in limit for AMI diagnosis) [38,39], it is important that these modified algorithms are prospectively validated in comparison with the algorithms recommended by the guidelines in a sufficiently large ED population before they are implemented in routine use.

### 3.2. Age-Adjusted Decision Limits in Rapid hs-cTn-Based Algorithms

By contrast, the published evidence supports the clinical impact of using age-adjusted decision limits in rapid algorithms for ruling-in ACS in elderly patients. The age-dependence of the URLs in healthy and apparently healthy elderly individuals has been convincingly demonstrated for cTnI and cTnT [30,36,37,40,41,42]. The reasons for this association are not fully understood, but subclinical cardiovascular and renal diseases are probably involved. The specificity of universal URLs is considerably lower than 99%, even for healthy elderly individuals. The hs-cTnT URL for elderly individuals >70 years is more than twice that of individuals <50 years [38]. The 0/1-h algorithm is significantly less effective among the elderly, >50% of patients were categorized in the “observe (gray) zone” [18,22,43].

Using higher age-adjusted decision thresholds has been demonstrated to provide clinical benefits for both cTnI and cTnT, with an improved positive predictive value for AMI in elderly patients who were classified as “rule-in” [31,43]. For patients ≥65 years, an age dependent cutoff twice as high as the uniform 99th percentile URL significantly reduces false-positive AMI classifications [31]. To improve the positive predictive value of the “rule-in” classification of cTnT and cTnI, markedly higher assay-specific baseline thresholds and slightly higher 1-h change thresholds are required, even in high-risk populations with suspected ACS [43].

**Figure 5:**
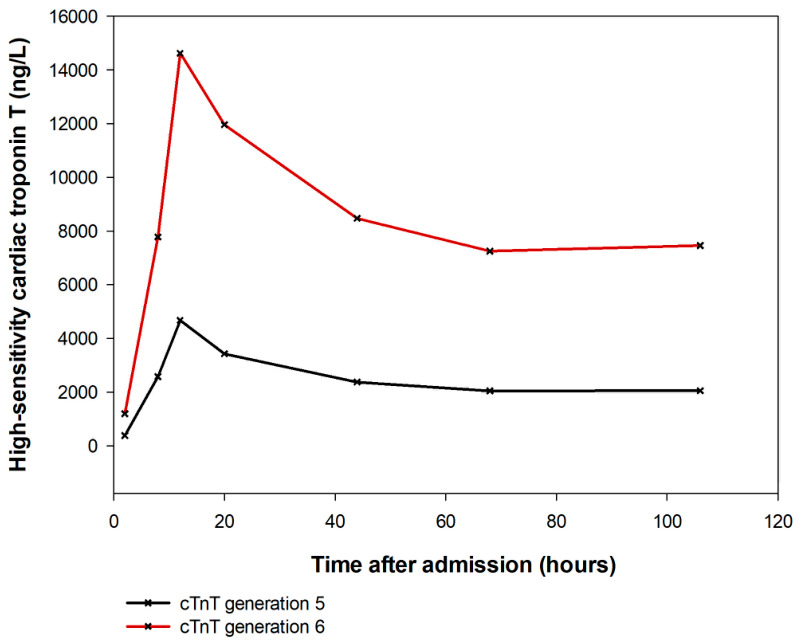
Cardiac troponin T concentration time courses tested with assay generation 5 (black) and 6 (red) in a patient with anterior wall ST-segment elevation myocardial infarction and successful primary percutaneous coronary intervention. Abbreviations: cardiac troponin T (cTnT).

Regarding the available, published, and adjusted cTnT decision limits, it should be noted that the data were obtained using the fifth generation of commercially available cTnT assays. However, with the introduction of the sixth generation of assays, it must be stressed that these published limits cannot be used anymore. Due to the differences in assay standardization (bovine versus human recombinant cTnT), the two assays correlate well, but do not have a 1:1 agreement in test results [44,45]. Depending on the measuring range of the fifth generation assay, the cTnT concentrations measured with the sixth generation assay are approximately 2- to 4-times higher (see Figure 5) [44,45]. There is no single, simple regression equation that is optimal across the entire measuring range.

### 3.3. Adjustment of Cardiac Troponin Decision Limits for Impaired Renal Function

Patients with chronic kidney disease are at a higher risk of developing ACS than the general population. Atypical clinical presentations without diagnostic ST-segment elevations but nonspecific repolarization abnormalities are common. However, an impaired chronic renal function is a major confounding factor that must be particularly considered when interpreting cTnT concentrations [46,47,48,49,50,51,52,53,54]. Although chronic increases in cTnI can also be found in this patient population, cTnT increases are much more common [46,47,48]. This does not impair the ability to rule out AMI, but it makes its rapid ruling in challenging. Therefore, changes in serial cTn measurements are particularly important for diagnosing AMI. As the glomerular filtration rate decreases, the hs-cTnT baseline and 2-h change values have to be markedly increased (2- to 6-fold depending on the stage of renal failure) to achieve an acceptable positive predictive value for AMI [49]. The reasons for the discrepancy between cTnI and cTnT in chronic renal failure are not yet fully understood [55], but it appears that, at low cTn concentrations, the kidneys have a greater impact on the clearance of cTnT from the circulation compared with cTnI [56,57]. It has been reported that smaller cTnT forms predominate in patients with chronic renal failure [58,59]. Conversely, cTnI is primarily found in the blood as part of complexes that are too large for glomerular filtration [60]. Currently, commercially available cTnI and cTnT assays are optimized to measure all circulating forms of either cTnI or cTnT to achieve high analytical sensitivity [55]. The reported optimal cutoff values for diagnosing AMI at ED presentation were 2- to 3-times higher in patients with even moderately impaired renal function, including cTnI [48,51]. Changes in cTn can help reach the correct diagnosis for most patients with renal failure; however, optimal delta values for ruling in AMI are higher [49,51,54]. Regardless of the baseline concentrations, cTn changes >2.5-fold over 3 h have a high positive predictive value for AMI, especially combined with ischemic ST-segment changes; however, overall, cTnI outperforms cTnT [51]. Nevertheless, in patients without AMI but with acute kidney injury, peak cTnT concentrations are associated with prognosis [61]. In summary, assessing cTn kinetics is essential for patients with chronic kidney diseases, and adopting higher cTn thresholds for ruling-in AMI and prospectively validating them will likely improve the overall diagnostic performance. Kidney function also affects the prognostic performance of cTnT and cTnI. The ideal cutoff for predicting mortality increases as the glomerular filtration rate decreases [54].

### 3.4. Clinical Challenge: Differential Diagnosis of Acute vs. Chronic Myocardial Injury in ED and Critically Ill Patients

Interindividual variability of cTn is greater than its intraindividual variability. Reference change values, considering both analytical and biological variability, are calculated to be >30% from baseline in follow-up testing [62]. Having an individual’s baseline cTnI or cTnT documented in the hospital information system is very useful for test result interpretation. With serial testing within 1 h to 3 h, biological variability may be minimal and can be neglected. Chronic myocardial injury (see Table 2) is defined as stable cTn elevations above the URL within analytical and biological variation. It is well-established that the higher the baseline cTn concentration and the greater the change in serial testing, the more likely is AMI in the clinical context of acute myocardial ischemia. The current criterion of a >20% change from baseline concentrations, which are higher than the URL, was defined to exclude analytical variation as a cause of cTn change in serial testing [1,3]. At that time, analytical variation is too high to reliably investigate biological variation, so the latter is not considered in this definition. However, when current state-of-the-art hs-cTn assays are used for cTn testing, which have a markedly improved analytical precision, and the time interval between sampling is only 1 h to 3 h, biological variation can be neglected, and the 20% threshold may be set too high. For example, with an hs-cTn assay that has a 10% coefficient of variation (CV) at 3 ng/L and a URL of 27 ng/L, the baseline value would be 30 ng/L. Considering only the analytical variation for baseline testing (CV = 3%), all follow-up concentrations after 1 h <27.5 and >32.5 ng/L (i.e., >±8.3% from baseline) are outside the analytical variation of baseline testing. Consistently, Lindahl et al. [63] recently reported that chronic myocardial injury cannot be reliably differentiated from acute myocardial injury by using the currently still recommended 20% change threshold over short remeasurement intervals in the ED. A small absolute value of 3 ng/L performed markedly better. However, there was still considerable overlap between patients with final diagnoses of acute and chronic myocardial injury, particularly among patients presenting very early or late after symptom onset (see Figure 3 and Figure 4) [63]. Therefore, it is difficult to definitively diagnose chronic myocardial injury using cTn testing during an ED stay. The consideration of all available clinical data, including imaging, is necessary, as is the frequently delayed follow-up cTn testing to confirm the diagnosis of chronic myocardial injury. Nevertheless, it is important to swiftly identify the most likely trigger of myocardial injury (e.g., tachyarrhythmia, severe anemia) to treat the underlying cause. In addition, Thießen et al. [64] derived a common rule-in/rule-out algorithm based on a 0/2 h sampling protocol that can be used with three frequently used routine hs-cTn assays from a high-risk chest pain population. They validated this algorithm in other high-risk study populations and found no significant difference in the performance of this algorithm when using established assay-specific absolute thresholds [8,9]. This algorithm is based on absolute values and relative changes, namely >3 ng/L for baseline values <10 ng/L, >30% change for baseline values between 10 ng/L and100 ng/L, and >15% change for baseline values >100 ng/L. However, the algorithm remains to be validated for the new sixth generation of the hs-cTnT assay.

There are numerous causes of acute myocardial injury in ED patients (see Table 1). AMI is defined as acute myocardial injury in a clinical setting that is consistent with acute myocardial ischemia, as demonstrated by typical ECG findings or imaging [1,2,3,4,5,6,7,8,9]. Thus, evidence of acute myocardial injury by significant cTn kinetics is necessary but not sufficient for the diagnosis of AMI. It is important to note that significant cTn kinetics indicating acute myocardial injury observed in ED patients (see Figure 1) is frequently unrelated to an acute coronary syndrome or acute myocardial oxygen supply/demand mismatch. Misclassifications are common, particularly in critically ill patients, where it is challenging to differentiate ischemic from non-ischemic acute myocardial injury. A symptom assessment and an ECG are unreliable, and cTn increases are found across a broad spectrum of diseases (see Table 1 and Table 2). Imaging to prove myocardial ischemia may not be a priority for acute management. Table 3 summarizes a practical approach for the work-up of such patients without typical ischemic ECG abnormalities. The cTn thresholds and kinetics during ED stay cannot reliably differentiate non-ST-segment elevation AMI from other causes of myocardial injury [65]. These rapid algorithms also perform worse in patients with known cardiac comorbidities, such as coronary artery disease, atrial fibrillation, or heart failure [19,20,66].

### 3.5. Interpretation of Cardiac Troponin in Symptomatic Athletes After Heavy Training Sessions or Competitions

**Figure 6:**
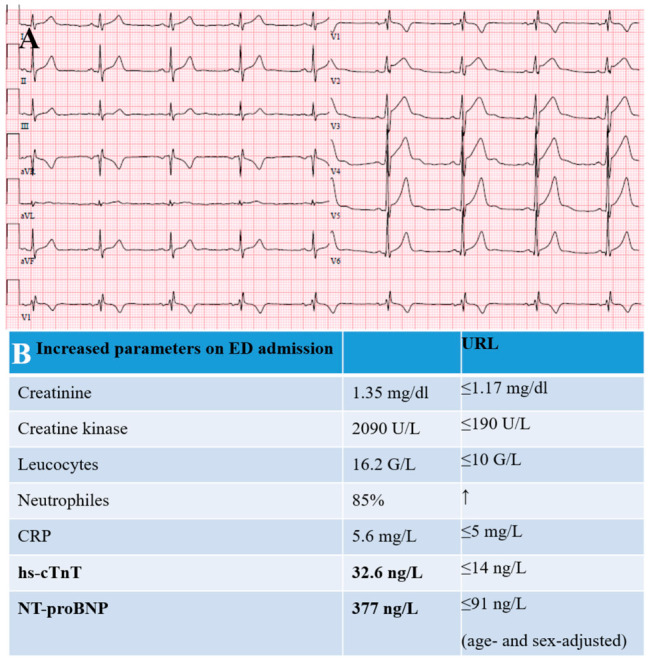
A clinical case demonstrating the challenges of troponin interpretation in symptomatic athletes after competitions or heavy training sessions. (**A**) Admission ECG; (**B**) Laboratory parameters upon admission. Abbreviations: high-sensitivity cardiac troponin T (hs-cTnT), N-terminal pro-B-type natriuretic peptide (NT-proBNP), C-reactive protein (CRP), upper reference limit (URL).

The interpretation of cTn in symptomatic athletes after heavy training sessions or competitions is rare, but it is particularly challenging (see Figure 6) [67,68,69]. Peak concentrations usually do not exceed 3-times the URL, and occur several hours after exercise. Concentrations return below the URL within 48–72 h [69]. A study simulating a marathon on a motorized treadmill, reported minimal cTnT release already within the first 60 min of running in a group of healthy, asymptomatic athletes [67], which suggests that exercise-induced cTn release is not restricted solely to prolonged endurance exercise. It is unlikely that these minor increases in cTn reflect myocardial necrosis in these individuals [70,71]. The frequent release of cTn in asymptomatic athletes complicates the interpretation of cTn concentrations in athletes who develop symptoms during or after competitions or heavy training sessions. Serial cTn testing, considering the magnitude of the cTn increase and rate of change, is essential to rule out acute myocardial ischemia as the cause of symptoms, particularly in older (>35 year old) male recreational athletes. In the absence of unequivocal ECG findings of acute myocardial ischemia, performing imaging, particularly coronary computed tomography angiography, is essential to rule out significant coronary artery disease.

This competition was an ultramarathon run (approximately 60 km). A 42-year-old male athlete who collapsed 400 m before reaching the finish line presented to the emergency department. He had known epilepsy that was very well controlled. Due to an abnormal resting ECG upon admission (A) and an increased high-sensitivity cardiac troponin T concentration (B), a computed tomography coronary angiography was performed. This examination excluded significant coronary artery disease and revealed a high-normal left ventricular ejection fraction (79%) with no regional wall motion abnormalities and an increased left ventricular stroke volume. The abnormal laboratory test results listed in (B) are typical for endurance athletes after finishing competitions. Although his ECG could not be compared with previous recordings, it was consistent with an incomplete right bundle branch block and early repolarization. The physical examination was unremarkable. He was asymptomatic during his stay in the emergency department and requested discharge after undergoing computed tomography.

## 4. Summary and Future Perspectives

Emergency department overcrowding has become a critical global health quality and patient safety issue. The introduction of rapid hs-cTn-based testing algorithms has transformed the management of patients with suspected ACS and has significantly impacted the length of stay in the ED. The 2-h algorithm offers practical advantages for implementation in the busy ED routine. It avoids the need to draw the second sample for cTn testing before knowing the baseline result and the challenge of sampling at 60 min after admission. An ED pathway based on these algorithms, including a structured clinical risk assessment, can reliably identify candidates for early ED discharge with outpatient work-up, if needed, based on the clinical probability of significant CAD. However, the positive predictive value of these algorithms for AMI in patients who are ruled-in is limited in a real-world ED setting. The clinical challenge lies in correctly classifying patients with cTn increases, for which a structured, stepwise approach is needed (see Table 1, Table 2 and Table 3). For rapid triage decisions in the ED, the definitive cause of acute myocardial injury has a limited impact because many of these patients require hospital admission for other reasons, even if their discharge diagnosis differs from AMI. However, for cardiologists, the positive predictive value of these pathways is critical for reliably identifying those patients who really need urgent or early invasive coronary angiography. The positive predictive value can be substantially improved by raising the cTn thresholds for elderly and in patients with chronic renal failure. However, this approach complicates the interpretation of cTn test results. Another challenge is managing ED patients with gray zone cTn concentrations. It can be difficult to differentiate between patients with acute and chronic myocardial injury with short-term serial testing, especially for early and late presenters. The initial criterion of a significant change of >20% in patients with baseline values less than the URL may be outdated with short-term serial testing due to marked improvements in the analytical precision of cTn assays since this criterion was initially defined. Additionally, biological variation may be neglected with a 1 h to 3 h sampling interval. Patients with chronic cardiac diseases or with chronic stable cTn elevations are a particular challenge when they present to the ED for diseases unrelated to their known cardiac disease. In this regard, cTn isoform testing could help to differentiate acute myocardial injury caused by AMI from other causes [72,73]. It has been reported that the so-called “long-forms” of cTnT are primarily found during the acute phase of AMI, while smaller forms are more prevalent in other etiologies, such as athletes or patients with chronic renal failure [58,59,69,70]. Promising data on discriminating ACS from other causes of myocardial injury have also been reported for calculating of cTnI/cTnT ratios [74]. However, the data must be verified for using the new sixth generation of the cTnT assay for cTnT measurement.

Artificial intelligence (AI)-assisted digital machine learning tools for evaluating patients with suspected ACS could be another helpful improvement to the daily routine [75,76,77]. These models estimate the individual probability of an AMI based on clinical information, ECG findings, and cTn test results. Still, a major limitation for ED physicians is that these tools are currently a “black box”, and users do not know on what basis decisions are made. Another critical issue is that the training data sets of these tools do not necessarily reflect the population of a real-world ED. Thus far, these tools have been trained using data sets from the clinical trials of preselected patients with clinically suspected ACS, and have been validated using data sets from other similar clinical studies. There is still, however, lack of sufficient performance data in real-world ED settings. It must be emphasized that all AI-based models require certification and registration as medical devices before routine use.

In summary, currently, a combined assessment of initial and/or serial hs-cTn testing, ECG findings, and clinical likelihood of significant CAD in a structured clinical pathway is still mandatory to identify those low-risk patients who can be discharged from the ED without follow-up investigations, those patients who require further outpatient work-up, and those patients who need urgent hospital admission.

## Figures and Tables

**Table 1:** Differential diagnosis of acute myocardial injury after exclusion of acute coronary syndrome and significant coronary artery disease.

1. Sustained tachy- or bradyarrhythmias. 2. Acute heart failure: e.g., cardiomyopathies, valvular heart diseases. 3. Acute inflammatory perimyocardial syndrome (myocarditis). 4. Sepsis. 5. Acute rejection after heart transplantation. 6. Hypertensive urgency/crisis. 7. Acute pulmonary embolism. 8. Toxic/metabolic: e.g., drugs (e.g., cocaine or amphetamine abuse), chemotherapy (e.g., Adriamycin, immune checkpoint inhibitors, 5-fluorouracil, trastuzumab), snake venom, burns. 9. Trauma: e.g., heart contusion, heart surgery, interventions (e.g., ablation, biopsy), multiple defibrillations/cardioversions. 10. Neurocardiac axis, massive activation of the sympathetic nervous system (likely etiology: myocardial microvascular perfusion disturbance): e.g., massive brain injury (e.g., ischemic stroke, hemorrhage, or trauma), Takotsubo (apical ballooning) syndrome. 11. Severe acute hypoxia: e.g., acute pulmonary decompensation, acute hemorrhage with or without shock, carbon monoxide intoxication. 12. Multifactorial causes: e.g., critically ill patients, grand mal seizures.

**Table 2:** Differential diagnosis of chronic myocardial injury.

1. Severe chronic coronary syndrome. 2. Chronic increased myocardial wall tension and/or myocardial overdistension: e.g., left ventricular hypertrophy, chronic heart failure (e.g., hypertrophic obstructive cardiomyopathy), severe valvular disease (e.g., aortic valve stenosis). 3. Severe chronic hypoxia: e.g., chronic pulmonary diseases (e.g., fibrosis, obstructive diseases, pulmonary hypertension), severe anemia. 3. Chronic inflammatory/immune-mediated cardiac diseases: e.g., myocarditis, sarcoidosis, rejection of the transplanted heart. 4. Deposits in the myocardium: e.g., amyloidosis, hemochromatosis. 5. Multifactorial causes: e.g., severe/end-stage chronic renal failure

**Table 3:** Proposed work-up for increased cardiac troponin concentrations in critically ill patients without ECG abnormalities typical of myocardial ischemia.

1. Confirm the presence of acute or chronic myocardial injury by assessing significant long-term troponin kinetics (>30% change from baseline). 2. Rule out structural heart disease and assess ventricular function using bedside echocardiography. 3. Rule out significant coronary artery disease after recovery by imaging, particularly in the elderly. 4. Identify and characterize the most likely alternative trigger of myocardial injury: e.g., shock, sustained tachycardia, hypertensive emergency, respiratory failure, sepsis. 5. Does troponin fall in response to treatment of the supposed trigger? 6. Perform additional imaging with cardiac magnetic resonance imaging if needed, e.g., for the definitive differentiation of ischemic from non-ischemic acute myocardial injury, confirmation or exclusion of myocarditis and rare structural heart diseases.

## Data Availability

No new data were created or analyzed in this study. Data sharing is not applicable to this study.
